# Gasdermin D deficiency attenuates arthritis induced by traumatic injury but not autoantibody-assembled immune complexes

**DOI:** 10.1186/s13075-021-02668-8

**Published:** 2021-11-16

**Authors:** Tong Yang, Kai Sun, Chun Wang, Gaurav Swarnkar, Songtao Quan, Dustin Kress, Jianqiu Xiao, Yael Alippe, Hongjun Zheng, Robert H Brophy, Dingjun Hao, Audrey McAlinden, Yousef Abu-Amer, Jie Shen, Gabriel Mbalaviele

**Affiliations:** 1 Xi’an Jiaotong University Health Science Center, Xi’an, Shaanxi China; 2 Division of Bone and Mineral Diseases, Washington University School of Medicine, 660 South Euclid Avenue, Campus Box 8301, St. Louis, MO 63110 USA; 3 Department of Orthopaedic Surgery, Washington University School of Medicine, St. Louis, MO USA; 4 Luoyang Orthopedic - Traumatological Hospital of Henan Province, Luoyang, Henan China; 5 Shriners Hospital for Children, St. Louis, MO USA

**Keywords:** GSDMD, IL-1, Inflammasome, Inflammation, Immune cells, Pyroptosis, Bone, Arthritis

## Abstract

**Background:**

Gasdermin D (GSDMD) is cleaved by several proteases including by caspase-1, a component of intracellular protein complexes called inflammasomes. Caspase-1 also converts pro-interleukin-1β (pro-IL-1β) and pro-IL-18 into bioactive IL-1β and IL-18, respectively. GSDMD amino-terminal fragments form plasma membrane pores, which mediate the secretion of IL-1β and IL-18 and cause the inflammatory form of cell death pyroptosis. Here, we tested the hypothesis that GSDMD contributes to joint degeneration in the K/BxN serum transfer-induced arthritis (STIA) model in which autoantibodies against glucose-6-phosphate isomerase promote the formation of pathogenic immune complexes on the surface of myeloid cells, which highly express the inflammasomes. The unexpected outcomes with the STIA model prompted us to determine the role of GSDMD in the post-traumatic osteoarthritis (PTOA) model caused by meniscus ligamentous injury (MLI) based on the hypothesis that this pore-forming protein is activated by signals released from damaged joint tissues.

**Methods:**

*Gsdmd* ^*+/+*^ and *Gsdmd*^*−/−*^ mice were injected with K/BxN mouse serum or subjected to MLI to cause STIA or PTOA, respectively. Paw and ankle swelling and DXA scanning were used to assess the outcomes in the STIA model whereas histopathology and micro-computed tomography (μCT) were utilized to monitor joints in the PTOA model. Murine and human joint tissues were also examined for GSDMD, IL-1β, and IL-18 expression by qPCR, immunohistochemistry, or immunoblotting.

**Results:**

GSDMD levels were higher in serum-inoculated paws compared to PBS-injected paws. Unexpectedly, ablation of GSDMD failed to reduce joint swelling and osteolysis, suggesting that GSDMD was dispensable for the pathogenesis of STIA. GSDMD levels were also higher in MLI compared to sham-operated joints. Importantly, ablation of GSDMD attenuated MLI-associated cartilage degradation (*p* = 0.0097), synovitis (*p* = 0.014), subchondral bone sclerosis (*p* = 0.0006), and subchondral bone plate thickness (*p* = 0.0174) based on histopathological and μCT analyses.

**Conclusion:**

GSDMD plays a key role in the pathogenesis of PTOA, but not STIA, suggesting that its actions in experimental arthropathy are tissue context-specific.

**Supplementary Information:**

The online version contains supplementary material available at 10.1186/s13075-021-02668-8.

## Background

Compelling evidence implicates inflammation in the pathogenesis of rheumatoid arthritis (RA), an autoimmune disease characterized by synovial inflammation and joint damage [1]. Although the etiology of RA remains unclear, its progression is associated with dysregulated inflammatory actions of various cell types including macrophages, neutrophils, T cells, B cells, and synovial fibroblasts [2–6]. These responses take place within the synovium and lead to the formation of the pannus, a tumor-like structure, which ultimately invades and damages the cartilage and bone [7, 8]. Several inflammatory cytokines contribute to joint pathology in RA, but the most significant include TNF-α, IL-1β, and IL-6, some of which partner with RANKL to promote osteoclast differentiation and, ultimately, osteolysis [9–11]. This premise is supported by the proven clinical efficacy of drugs that neutralize the activity of these cytokines [12–17]. However, RA is a complex disease with superimposed actions of cytokines and autoantibodies, including rheumatoid factor, anti-citrullinated, and carbamylated protein autoantibodies [2–6]. As a result, certain patients are refractory to current cytokine-based therapies, and in spite of the pivotal role of IL-1β in maintaining synovitis and inducing bone erosion, IL-1 blockade shows limited efficacy in improving clinical symptoms of RA patients [15].

In contrast to RA, OA was historically deemed as a simple process of cartilage deterioration. However, the pathogenesis of OA is now viewed as a complex process, which depending on the etiology (e.g., caused by trauma) is attended by subchondral bone sclerosis, synovitis, osteophyte formation, and inflammation [18]. Dysregulated inflammatory networks include those governed by IL-1 family members such as IL-1α, IL-18, and IL-1β [19, 20], which are potent inducers of tissue-degrading enzymes such as matrix metalloproteases (MMPs) and a disintegrin and metalloproteinase with thrombospondin motifs (ADAMTS) [21, 22]. However, the role of IL-1 in OA remains inconclusive as both joint-protective and joint-damaging effects of IL-1 signaling blockage have been reported in various pre-clinical models [23, 24]. More importantly, several clinical trials of OA have reported a limited efficacy of IL-1 biologics [25, 26]. In the absence of efficacious disease-modifying OA drugs, surgical approaches such as joint replacement are by far the most effective interventions to control pain and correct joint malformation of late-stage OA patients. The high costs and invasive nature of these procedures justify the search for novel targets in the therapeutic intervention of OA.

Similar to IL-1β and IL-18, the gasdermin family member, GSDMD, is activated upon cleavage by caspase-1, a component of the inflammasomes, which are intracellular protein complexes that are assembled upon detection of pathogenic signals and sterile stressors [27]. GSDMD amino-terminal fragments oligomerize and form pores at the plasma membrane through which IL-1β and IL-18 are secreted as they lack the signal peptide for the transport through the conventional endoplasmic reticulum-Golgi pathway [28]. However, excessive GSDMD pore formation compromises the integrity of the plasma membrane and leads to lytic cell death pyroptosis [28]. Pyroptosis uncontrollably releases cell contents, which include not only the active form of IL-1β and IL-18 but also other highly inflammatory danger-associated molecular patterns (DAMPs) such as ATP, high mobility group box 1 (HMGB1), and S100A9 proteins [29]. The release of these DAMPs results in further recruitment of immune cells and the perpetuation of inflammation.

GSDMD is an attractive target for therapeutic intervention; this view stems from the demonstration that loss of this protein prevented the pathogenesis not only of autoinflammatory monogenic disorders such as neonatal-onset multisystem inflammatory disease (NOMID) and familial Mediterranean fever (FMF) in mice, but also complex diseases such as experimental autoimmune encephalitis (EAE) and sepsis [30–33]. Since GSDMD pores facilitate not only the secretion of IL-1β, IL-18, and other small molecules by live cells (aka hyperactive cells) but also various inflammatory intracellular contents during pyroptosis, inhibition of GSDMD pore-forming activity could, in theory, provide superior efficacy over IL-1 blockade. However, this view is not universal as a recent paper reported that deletion of GSDMD had no impact on monosodium urate-induced gouty arthritis in mice [34]. A conflicting role of GSDMD in dextran sodium sulfate (DSS)-induced colitis in mice has also been reported [35, 36]. Thus, the extent to which inhibition of GSDMD prevents the pathogenesis of complex diseases such as RA and OA has yet to be established.

In this study, we sought to determine the role of GSDMD in arthritis using mice sufficient or insufficient in this protein. We found that GSDMD deficiency attenuates arthropathy induced by traumatic injury but not immune complexes assembled by autoantibodies.

## Materials and methods

### Human tissues

Synovial tissues were collected from eight late-stage OA patients (5 females, 3 males) with ages ranging from 56 to 74 years during total knee replacement (TKR) surgery and one healthy patient who had a bone fracture, during fixation removal surgery. The criteria for TKR included joint pain that lasted over 12 months with evidence of radiological changes including moderate joint space narrowing and osteophytes formation. OA cartilage was collected during total knee arthroplasty (TKA) from terminal OA patients whereas PTOA cartilage was obtained from patients who developed clinical signs of knee OA after anterior cruciate ligament (ACL) injury. Control cartilage was from areas with normal cartilage appearance from the same patients. The collection of tissues was approved by the review board of Washington University School of Medicine in St. Louis. All participants provided written informed consent.

### Mice

*Gsdmd* knockout (*Gsdmd*^*−/−*^) mice, kindly provided by Dr. Vishva M. Dixit (Genentech, South San Francisco, CA), were generated using CRISPR-Cas9 technology [33]. All mice were backcrossed to the C57BL6 strain for at least 10 generations, and mouse genotyping was performed by PCR. All procedures were approved by the Institutional Animal Care and Use Committee (IACUC) of Washington University School of Medicine in St. Louis.

### Serum transfer-induced arthritis (STIA)

Six-week-old *Gsdmd*^*+/+*^ and *Gsdmd*^*−/−*^ mice were injected with K/BxN mouse serum (150 μl) intraperitoneally on days 0 and 2 as described previously [37]. Mice inoculated with PBS served as controls. Mice were monitored daily after injections. Paw and ankle thicknesses were measured daily for 12 days with a digital caliper. Tissues were collected on day 12 for further analysis.

### Meniscal ligamentous injury (MLI) model

Twelve-week-old *Gsdmd*^*+/+*^ and *Gsdmd*^*−/−*^ male mice were subjected to MLI surgery [38]. Briefly, the medial collateral ligament was transected, then a portion of the anterior medial meniscus was surgically removed without disrupting the patella and any other ligaments. Sham surgery was performed on the contralateral joint of the same mouse in which a similar incision is made on the medial side without the removal of the meniscus or the collateral ligament. Mice were sacrificed 12 weeks afterwards, and joint tissues were collected.

### Histology and immunochemistry

The knee joints were fixed in 10% neutral buffered formalin at room temperature for 24 h then decalcified in Immunocal (StatLab, McKinney, TX) for 3 days; fresh Immunocal was changed every 24 h. Tissues were processed and paraffin-embedded, then 5-μm-thick sagittal sections were generated, starting from the medial side of the knees. They were stained with Safranin-O. OARSI scoring was based on an established scoring system [39]. Synovitis scoring was based on the severity of synovium hyperplasia (score range 0–3) and inflammatory cell infiltration (score range 0–3) in the sub-synovial region [40]. All histological scoring was performed by two blinded reviewers.

For immunohistochemistry, the sections were deparaffinized and rehydrated using xylene followed by an ethanol gradient. Antigen retrieval was performed using citrate buffer (Dako, S1699) at 3 psi. The sections were incubated overnight at 4 °C with primary antibodies against GSDMD (1:200, Abcam, ab219800), followed by 1 h incubation with biotinylated goat secondary antibody (Vector Laboratories, BA-9200). DAB reagent (Vector Laboratories, SK-4100) was used for the peroxidase-substrate reaction.

### Micro-computed tomography

Before decalcification, the knee joints were scanned at a resolution of 10 μm, 55 kVp, 145 μA, and 300 ms integration time, using a micro-computed tomography system (μCT 40 scanner; Scanco Medical AG, Zurich). Bone volume/total volume (BV/TV) and subchondral bone plate thickness of medial tibial subchondral bones were analyzed using the Scanco analysis software. 3D images were reconstructed using the Dragonfly software (Dragonfly 3.6, Object Research Systems (ORS) Inc., Montreal, Canada). A “PET” color scheme was used instead of the conventional black and white to highlight the subchondral bone changes.

### Scanning of the limbs

The hindlimbs were scanned with Faxitron UltraFocus DXA (Hologic Inc., MA, USA). 3D images were reconstructed using Dragonfly software (Dragonfly 3.6, Object Research Systems (ORS) Inc., Montreal, Canada).

### Cell culture and treatment

Murine primary bone marrow-derived macrophages (BMDMs) were obtained using the bone marrow from the femurs and tibiae in MEM-α (Gibco, 12561-049) with 10% FBS (Gibco, 26140-079) and 1% Pen/Strep (Gibco, 15140-122) containing 10% CMG, a source of M-CSF, for up to 5 days in a 150-mm dish. After expansion, BMDMs were plated at a density of 5× 10^4^, 1 × 10^6^ cells/well in a 16-well chamber slide, or 60 mm tissue culture dish, respectively. For inflammasome activation in vitro, BMDMs were primed with 100 ng/ml LPS (Sigma Aldrich, L4391) or 20 ng/ml TNF-α (R&D systems, aa 80-235) for 3 h, then followed with 15 μM nigericin (Sigma Aldrich) for 1 h.

Primary articular chondrocytes were isolated from murine femoral heads. Cells were harvested from the cartilage digested with collagenase P (0.5 mg/ml, Roche, 11249002001) for 4 h. Cells seeded at a density of 1 × 10^6^ and 1 × 10^5^ cells/well in 6-well plate or 16-well chamber slide, respectively, were treated with 1 ng/ml IL-1β for 24 h.

### qPCR

#### Mice

RNA was isolated from whole paws 12 days after PBS or K/BxN serum injection or femoral and tibia articular cartilage 4 weeks after sham or MLI surgery.

#### Humans

RNA was harvested from the articular cartilage from controls or patients with OA or PTOA. Total RNA was isolated using the PureLink RNA mini kit (Invitrogen). cDNA was synthesized using the iScript reverse transcription kit (Bio-Rad Laboratories) then amplified using *Gsdmd/GSDMD*, *Gsdme*, *IL1B*, or *IL18* primers (Supplementary Table 1). Gene expression was analyzed by qPCR using the SYBR green gene expression assay (Applied Biosystems).

### Western blot

Cells and tissues were lysed with RIPA buffer (50 mM Tris, 150 mM NaCl, 1 mM EDTA, 0.5% NaDOAc, 0.1% SDS, and 1.0% NP-40) plus Complete Protease Inhibitor Cocktail and phosphatase inhibitors (Roche, South San Francisco, CA, USA). Protein concentrations from cell lysates and tissue lysates were determined by the Bio-Rad method. Proteins were separated by SDS-PAGE (12%) and transferred to PVDF membranes, which were incubated with antibodies against mouse GSDMD (1:1000, ab219800, Abcam), human GSDMD (1:1000, ab210070, Abcam), or β-actin (1:5000, sc-47778, Santa Cruz Biotechnology, Dallas, TX, USA) overnight at 4 °C, followed by a 1-h incubation with secondary goat anti-rabbit IgG (1:5000, A21109, Thermo Fisher Scientific, Waltham, MA, USA). The signals were developed using the Li-Cor Odyssey Infrared Imaging System (LI-COR Biosciences, Lincoln, NE, USA).

### Measurements of IL-1β and lactate dehydrogenase (LDH)

Cell death was assessed by the release of LDH in a conditioned medium using the LDH Cytotoxicity Detection Kit (TaKaRa, CA). IL-1β levels in conditioned media were measured by ELISA (eBiosciences, NY).

### Statistical analysis

Statistical analysis was performed using repeated measures ANOVA and *t* test analysis based on the data type (described in the figure legends) in GraphPad Prism (version 8.0.0, GraphPad Software, San Diego).

## Results

### GSDMD deficiency failed to reduce joint swelling and osteolysis in the STIA model

**Fig. 1:**
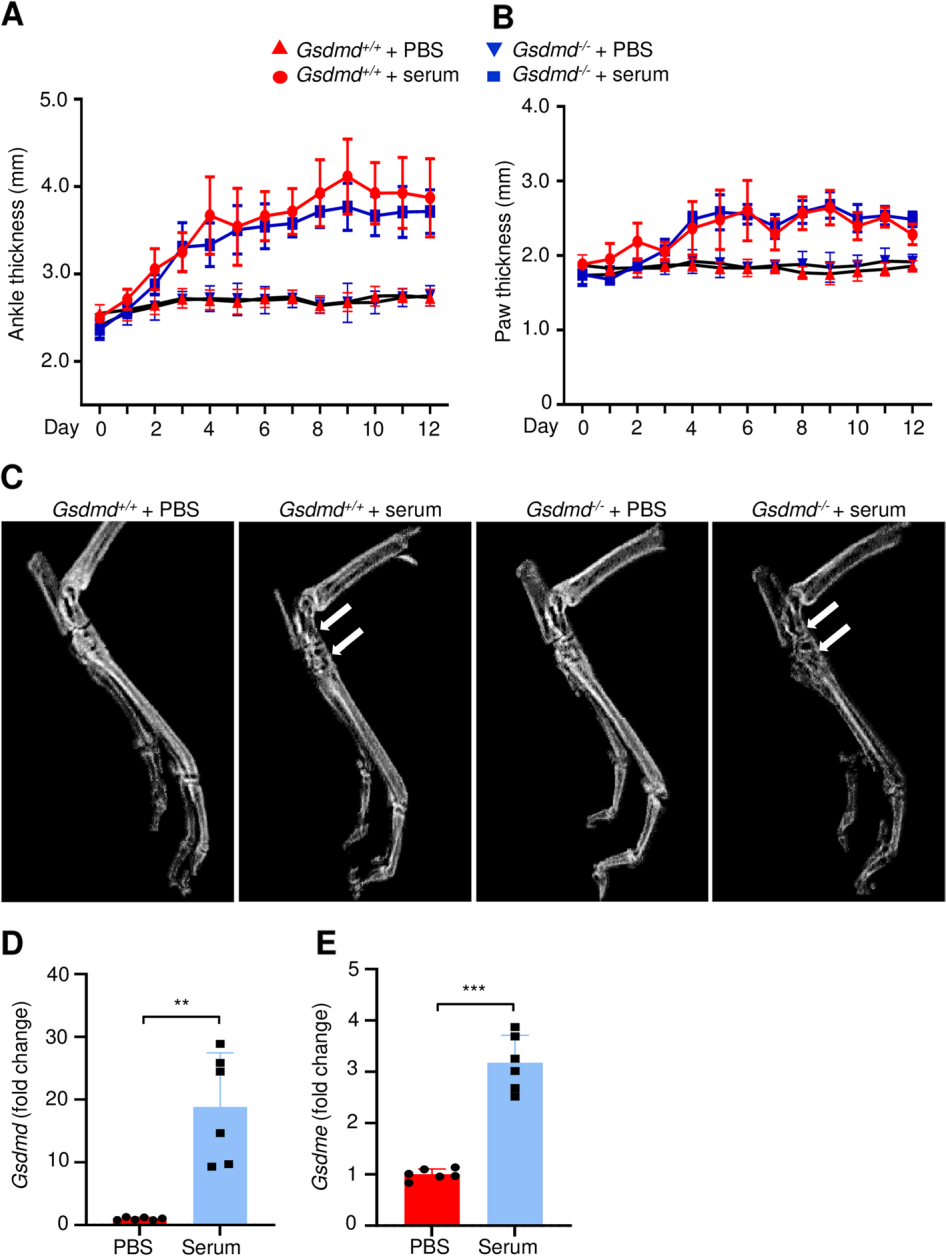
GSDMD deficiency did not reduce joint swelling and osteolysis induced by STIA. Six-week-old mice were injected intraperitoneally with PBS (*Gsdmd*^*+/+*^: 4 females and 3 males; *Gsdmd*^*−/−*^: 4 females and 2 males) or K/BxN mouse serum (*Gsdmd*^*+/+*^: 3 females and 2 males; *Gsdmd*^*−/−*^: 4 females and 3 males) on days 0 and 2. Ankle (**A**) and paw (**B**) thicknesses were measured daily for 12 days with a digital caliper. No differences in joint swelling were noted between male and female mice. **C** The hindlimbs were collected on day 12 and scanned. White arrows indicate the areas of extensive osteolysis. **D**, **E** qPCR analysis of *Gsdmd* mRNA expression in *Gsdmd*^*+/+*^ paw tissues. Data are mean ± *SD*. Student’s *t* test; **p < 0.01; ***p < 0.001

The serum of K/BxN mice contains autoantibodies against glucose-6-phosphate isomerase (G6PI), which upon transfer to recipient animals consistently cause inflammatory arthritis [37]. This model commonly referred to as serum transfer-induced arthritis (STIA) reproduces the effector phase of RA as G6PI autoantibodies promote the formation of immune complexes that activate innate immune cells such as neutrophils and macrophages. The prominence of inflammasome pathways in myeloid cells, which play key roles in the pathogenesis of STIA, provided a strong rationale for determining the joint impact of GSDMD deficiency in this model. Inoculation of the arthritic serum but not PBS caused the swelling of the ankles (Fig. 1A; Fig. S1A) and paws (Fig. 1B) in *Gsdmd*^*+/+*^ mice. These joint outcomes were unaffected in mice lacking GSDMD (Fig. 1A, B; Fig. S1B). Likewise, bone destruction at the tibial-tarsal junctions was comparable between *Gsdmd*^*+/+*^ and *Gsdmd*^*−/−*^ mice (Fig. 1C). These results suggest that GSDMD does not play a key role in the pathogenesis of STIA.

The unexpected comparable phenotype of *Gsdmd*^*+/+*^- and *Gsdmd*^*−/−*^ -serum injected mice prompted us to analyze the expression of *Gsdmd* mRNA in the paws in this model. We found that paws from serum-treated WT mice expressed high levels of *Gsdmd* transcripts compared to PBS-treated controls (Fig. 1D). Levels of GSDME, another widely studied GSDM family member [41], were also higher in STIA paws compared to PBS counterparts (Fig. 1E). Thus, although GSDMD expression is induced by the antibodies-containing serum, its absence does not attenuate the associated inflammation and osteolysis.

### GSDMD expression was upregulated in PTOA joints

**Fig. 2:**
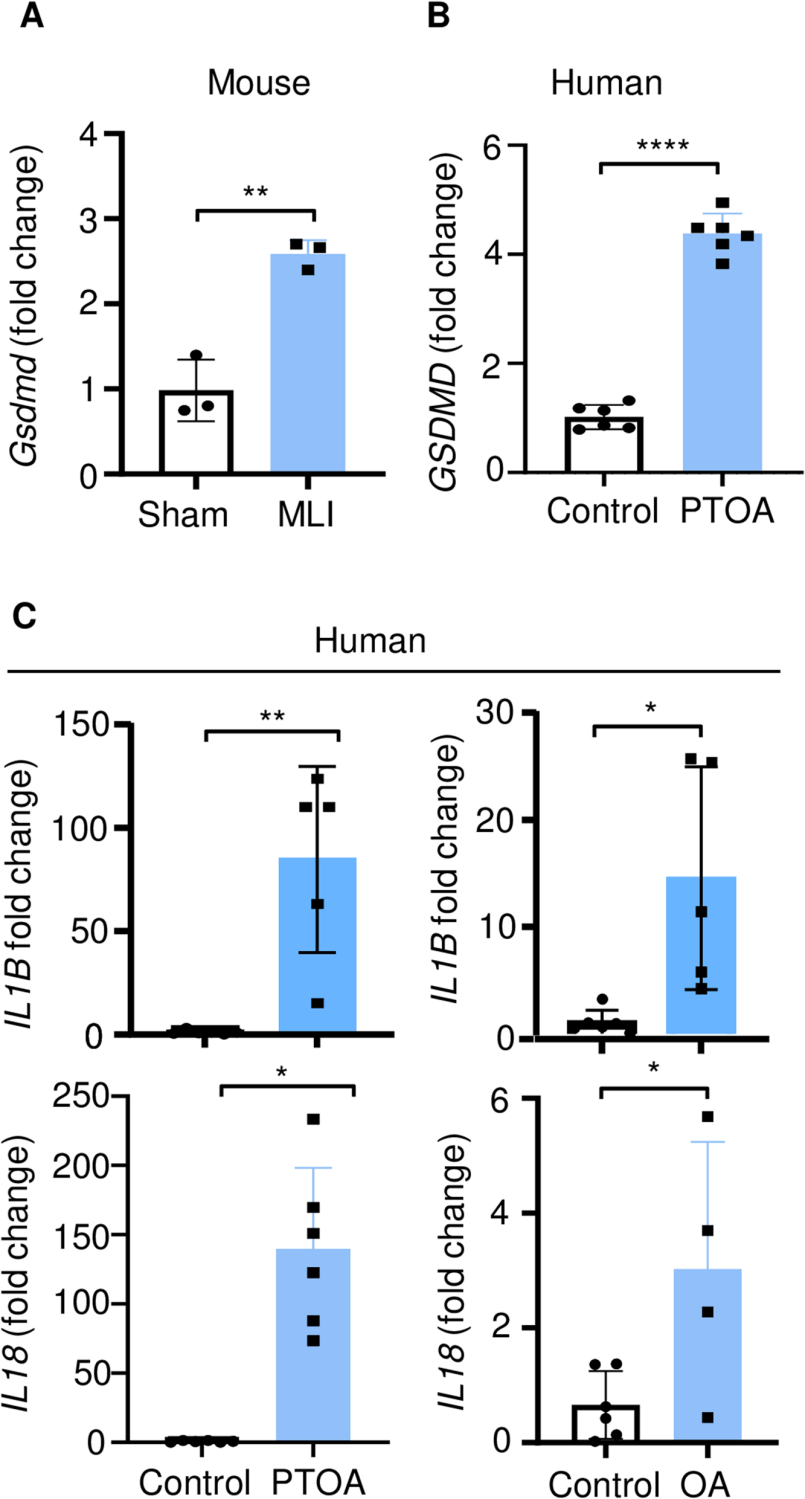
Expression of GSDMD, IL-1β, and IL-18 was increased in the articular cartilage from mice subjected to MLI and human PTOA patients. qPCR analysis of the gene expression in the articular cartilage from mice (sham surgery or MLI (**A**)) and human patients (control, OA, or PTOA (**B**, **C**)). The dot points reflect different patients or mouse samples. Data are mean ± *SD*. Student’s *t* test; *p < 0.05; **p < 0.01; ****p < 0.0001

Inhibition of inflammasomes, which are involved in the activation of GSDMD, prevents OA pathogenesis in various animal models [42–45]. Therefore, we determined the impact of GSDMD deficiency on the joints in the PTOA model induced by meniscal ligamentous injury (MLI). First, we analyzed GSDMD expression in the articular cartilage. The articular cartilage from MLI mice expressed higher levels of *Gsdmd* mRNA compared to sham controls (Fig. 2A). We also analyzed the expression of *GSDMD*, *IL1B*, and *IL18* mRNA in human articular cartilage. The levels of *GSDMD* (Fig. 2B) and *IL1B* and *IL18* (Fig. 2C) transcripts were higher in human PTOA or OA articular cartilage compared to control counterparts. *GSDMD* expression was also increased in OA articular cartilage compared to control cartilage (Fig. S2A). Thus, GSDMD expression is upregulated in osteoarthritic articular cartilage.

**Fig. 3:**
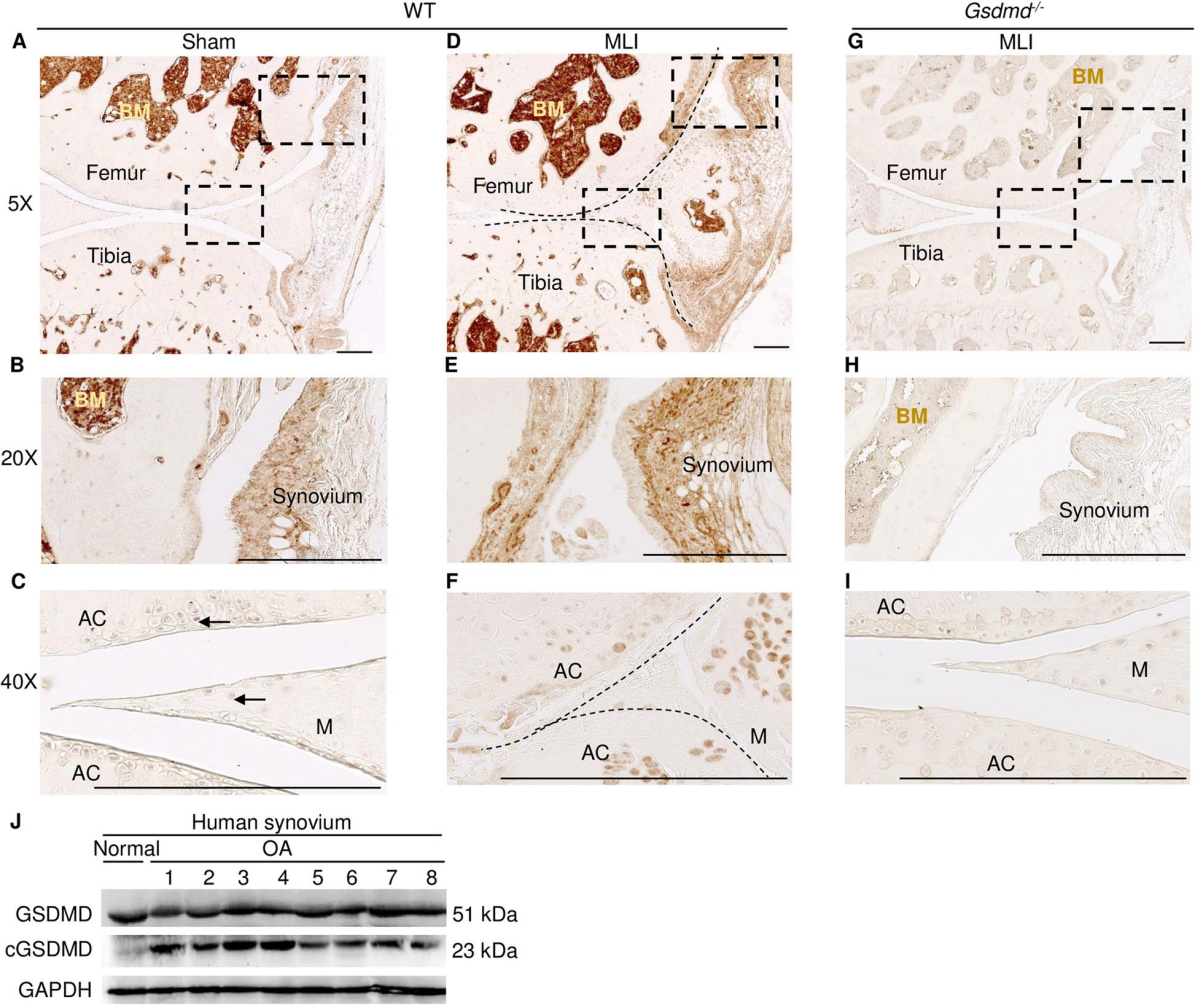
GSDMD was expressed in mouse joint tissues and synovium of human OA patients. **A**–**F** Representative images of specimens of *Gsdmd*^*+/+*^ mice stained with GSDMD antibody. **B**, **C**, **E**, **F** Magnified views of the boxed areas in **A** and **D**. **G**–**I** Representative images of specimens of *Gsdmd*^*−/−*^ mice stained with GSDMD antibody. Brown and dark red show specific staining. Scale bar = 250 μm (**A**–**I**). AC, articular cartilage; BM, bone marrow; M, meniscus. **J** Immunoblotting analysis of GSDMD expression and cleavage in the synovium from 1 normal and 8 OA patients. GAPDH was used as a loading control

To further analyze GSDMD protein expression in the cartilage and surrounding tissues as well, specimens from the whole joint were immunostained with GSDMD antibodies. Immunohistochemical analysis showed that GSDMD was expressed in the bone marrow and synovium but was barely detected in the articular cartilage and meniscus in sham-operated knees (Fig. 3A–C). GSDMD expression persisted in the synovium and was induced in the meniscus and articular cartilage in the hind limbs subjected to MLI (Fig. 3D–F). The absence of the immunostaining in *Gsdmd*^*−/−*^ tissues validated the specificity of the antibody (Fig. 3G–I). We also leveraged the immunoblotting method to determine the expression and activation state of GSDMD in the synovium of human patients. We found that GSDMD was cleaved in all synovium of OA patients that we analyzed (Fig. 3J). Collectively, the results show that GSDMD is expressed in several joint tissues including the cartilage, meniscus, and synovium.

Within the arthritic joints, cytokines such as TNF-α and IL-1β act on various cell types including macrophages [19, 46]. For proof-of-concept studies, we determined the role of GSDMD in TNF-α-induced production of IL-1β using macrophages from bone marrow instead of arthritic tissues. Exposure of murine macrophages primed with TNF-α or LPS (positive control) to the ionophore nigericin, which induces NLRP3 inflammasome assembly [30] resulted in the cleavage of GSDMD (Fig. S2B), IL-1β secretion (Fig. S2C), and the release of lactate dehydrogenase (LDH), a readout of pyroptosis (Fig. S2D). These responses were all blunted in macrophages lacking GSDMD and are consistent with the known role of GSDMD in the propagation of inflammation.

### GSDMD deficiency attenuated the loss of articular cartilage and synovitis in the murine PTOA model

**Fig. 4:**
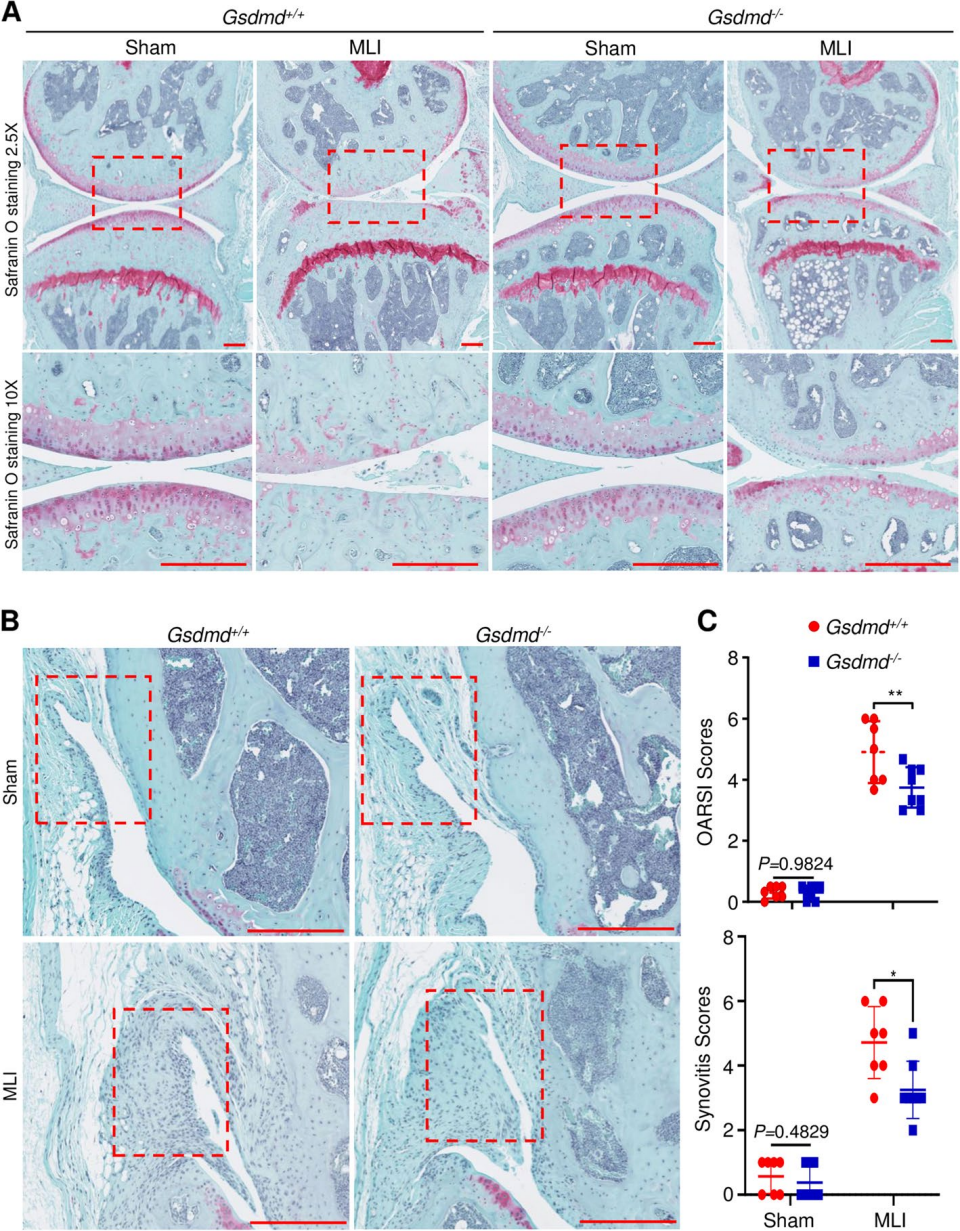
GSDMD deficiency attenuated articular cartilage degeneration and synovitis. Twelve-week-old *Gsdmd*^+/+^ and *Gsdmd*^*−/−*^ male mice were subjected to sham or MLI surgery. **A** Representative images of Safranin-O staining of the left (sham) and right (MLI) knee joints. **B** Representative images of anterior synovium of sham or MLI knee joints from *Gsdmd*^+/+^ and *Gsdmd*^*−/−*^ mice. **C** OARSI and synovitis scores. OARSI scoring was performed to quantify the severity of OA. Synovitis scores are based on the severity of synovial hyperplasia and sub-synovial inflammation. Reference data for **C** are shown in Fig. S3A. Data are mean ± *SD*. *N* = 7–8/group. Unpaired *t* test; *p < 0.05; **p < 0.01. Scale bar = 250 μm

To determine the impact of GSDMD deficiency on a joint response to injury, *Gsdmd*^*+/+*^ and *Gsdmd*^*−/−*^ mice were subjected to MLI or sham surgery. Histological examinations of Safranin O-stained specimens revealed that articular cartilage and growth plates were morphologically comparable between sham-operated *Gsdmd*^*+/+*^ and *Gsdmd*^*−/−*^ knees (Fig. 4A), suggesting that this protein did not impact the development of these skeletal structures. MLI induced a marked loss of articular cartilage in *Gsdmd*^*+/+*^ mice compared to *Gsdmd*^*−/−*^ animals (Fig. 4A). This outcome was strongly correlated with OARSI scores, which were high in *Gsdmd*^*+/+*^ compared to mutant mice (Fig. 4C and Fig. S3A). MLI also induced synovitis, which was more severe in *Gsdmd*^*+/+*^ compared to *Gsdmd*^*−/−*^ mice (Fig. 4B, C). Thus, GSDMD deficiency attenuates cartilage dedegeneration and synovitis in the PTOA model.

### GSDMD deficiency attenuated subchondral bone sclerosis in the murine PTOA model

**Fig. 5:**
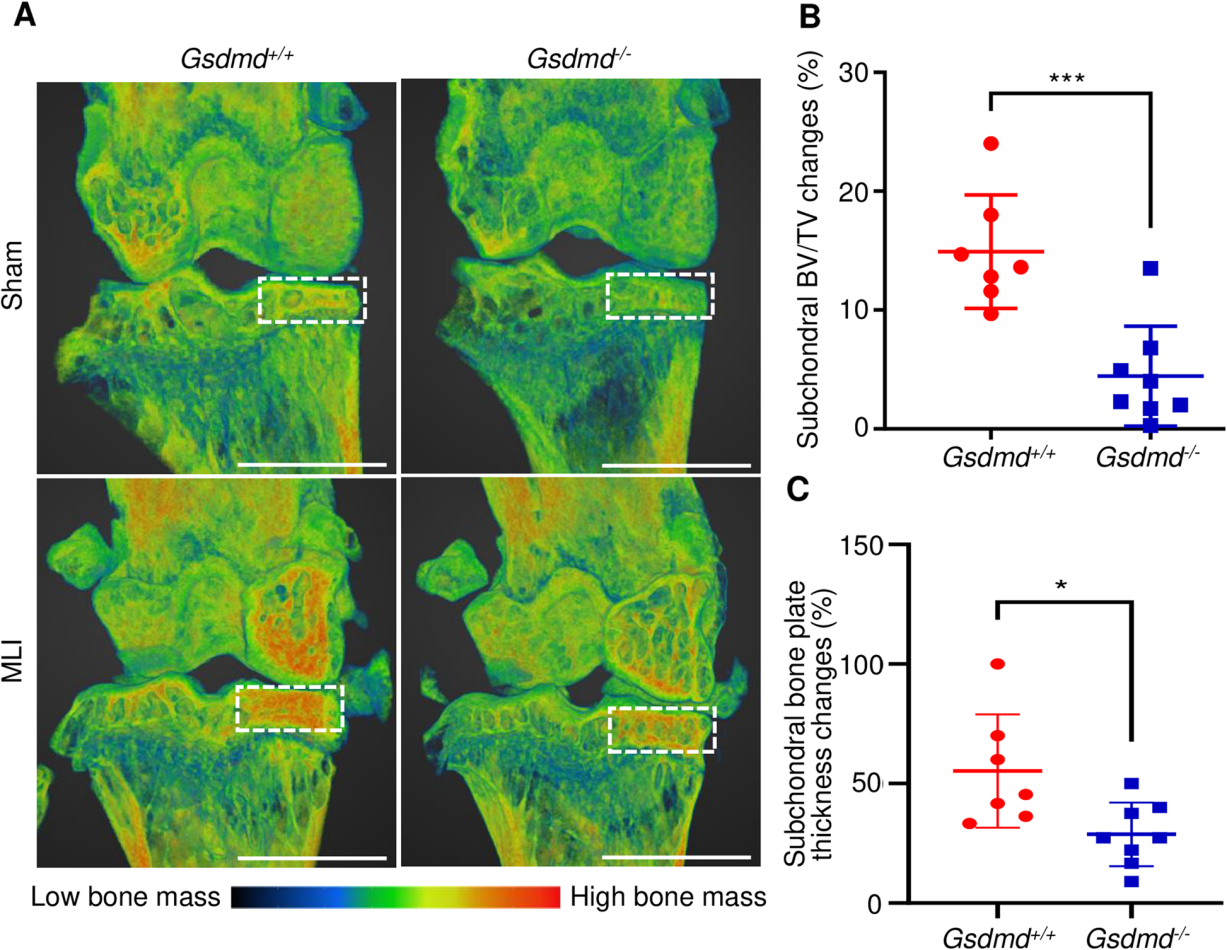
GSDMD deficiency attenuated subchondral bone sclerosis. Twelve-week-old *Gsdmd*^+/+^ and *Gsdmd*^*−/−*^ male mice were subjected to sham or MLI surgery. **A** Representative three-dimensional μCT figures of sham and MLI knee joints. “PET” color scheme from the Dragonfly software was used to highlight the subchondral bone changes. **B** Subchondral BV/TV changes. MLI BV/TV/Sham BV/TV percentage for each mouse was used to quantify the extent of subchondral bone sclerosis. Reference data are shown in Fig. S3B. **C** Subchondral bone plate thickness changes. MLI thickness/sham thickness percentage for each mouse was used to quantify the extent of subchondral bone sclerosis. Data are mean ± *SD*. *N* = 7–8/group. Unpaired *t* test; *p < 0.05; ***p < 0.001. Scale bar = 5 mm

MLI perturbs various tissues in the joint, including subchondral bone [47]. To understand the role that GSDMD plays in subchondral bone sclerosis, we used μCT combined with a PET color scheme, instead of the conventional black and white modality, for visual examinations of bone changes. 3D reconstruction images showed that baseline bone morphology was comparable between sham-operated *Gsdmd*^*+/+*^ and *Gsdmd*^*−/−*^ mice (Fig. 5A). Focusing on the joint medial side, which is consistently affected in the MLI model [47], we found that the injury led to subchondral bone accrual in *Gsdmd*^*+/+*^ hind limbs, a response that was reduced in *Gsdmd*^*−/−*^ counterparts (Fig. 5A). Although subchondral BV/TV was slightly elevated in mutant compared to *Gsdmd*^*+/+*^ tibia (Fig. S3B), BV/TV and subchondral bone plate thickness changes between MLI and sham tibial plateaus were higher in *Gsdmd*^*+/+*^ bones compared to *Gsdmd*^*−/−*^ counterparts (Fig. 5B, C). These results suggest that lack of GSDMD attenuates the pathogenesis of subchondral bone sclerosis in MLI-induced OA.

## Discussion

We found that GSDMD was not essential for the pathogenesis of STIA. This finding from a model in which the effector phase of arthritis was driven by myeloid cells was unexpected because these cells express high levels of GSDMD [41]. In addition, loss of GSDMD was shown to prevent tissues from damage in disease models of EAE, NOMID, IBD, sepsis, and FMF [30–33]. We postulate that the unanticipated outcome of GSDMD deficiency in the STIA model may be the result of redundancy among GSDM family members. GSDME was the likely culprit because of its ability to complement GSDMD functions [41, 48–50], and, like GSDMD, its levels were higher in STIA paws compared to PBS paws. Non-essential or conflicting roles for several proteins that operate upstream or downstream of GSDMD had also been reported in models of inflammatory arthritis. Indeed, mice lacking the inflammasome adaptor protein apoptosis-associated speck-like protein containing a CARD (*Asc*) but not *Nlrp3* or caspase-1 mice were protected from collagen-induced arthritis and antigen-induced arthritis [51, 52] whereas NLRP3 and IL-1 receptor type 1 (IL-1R1) were important for the pathogenesis of arthritis induced by A20 ablation [53]. Furthermore, the lack of IL-1 signaling in mice suppressed CIA-driven arthritis [54]. Thus, not only GSDMD, but also several components of its signaling pathway are dispensable in animal models of autoimmune arthritis.

The development of PTOA caused by MLI is associated with increased levels of pro-inflammatory cytokines such as IL-1β and TNF-α, whose downstream targets include matrix-degrading enzymes such as MMPs and aggrecanases [21, 22]. Consistent with the pro-inflammatory actions of GSDMD, MLI-induced OA is significantly attenuated in mice lacking this pore-forming protein. Thus, besides NOMID, FMF, EAE, and sepsis, PTOA is another experimental disease model in which ablation of GSDMD attenuates tissue damage [30–33]. These genetic proof-of-concept studies provide a rationale for testing the efficacy of GSDMD inhibitors such as FDA-approved disulfiram and dimethyl fumarate in OA pathogenesis [55, 56]. Although blocking specific components of the inflammasome pathways such as IL-1β provides limited efficacy in OA, the expectation is that by preventing the release of not only IL-1β and IL-18 but also pyroptosis, inhibition of GSDMD may provide superior efficacy over IL-1 blockade [30].

GSDMD deficiency attenuated not only cartilage deterioration but also subchondral bone sclerosis, consistent with the bone-cartilage interplay during the pathogenesis of OA [57]. Subchondral bone sclerosis correlates with decreased bone remodeling and is believed to promote the initiation and progression of OA as it creates a stiff structure that promotes cartilage degeneration [57]. This dogma is consistent with our results though baseline subchondral bone mass is higher in GSDMD-deficient mice compared to WT littermates. Mechanistically, we recently reported that metaphyseal trabecular bone mass is increased in GSDMD-deficient mice as the result of decreased osteoclast differentiation [58]. However, efforts to understand whether bony changes precede cartilage alterations or vice versa are constrained by the uneasy access to *floxed-Gsdmd* mice for tissue-specific ablation of GSDMD.

GSDMD was expressed in the synovium. Although the characterization of GSDMD^+^ cells in the synovium was outside the scope of this study, we posited that they included macrophages, which populate this tissue in various arthritis disease models [19, 46, 59] and highly express GSDMD [30, 41, 58]. Within the synovium, GSDMD activation by cleavage is likely induced by cytokines and inflammasome assembly molecules such ATP and debris from dying cells or damaged tissues [60, 61]. We found that GSDMD was cleaved in the samples from OA patients. However, the limitation of this observation was the challenge of obtaining several healthy synovial tissues for rigorous comparison of the extent of GSDMD cleavage between normal and OA samples. Despite this shortcoming, we contend that the actions of GSDMD in the synovium and other tissues such as the meniscus may culminate in cartilage deterioration in PTOA. Cartilage catabolic actions of GSDMD may also be chondrocyte autonomous as these cells express this protein. The ability of chondrocytes to upregulate GSDMD expression upon exposure to IL-1β (Fig. S4) suggests autocrine events whereby this cytokine acts locally on these cells to perpetuate cartilage demise.

## Conclusions

In conclusion, GSDMD was induced in the cartilage and synovium of OA patients and mouse model of this disease. Importantly, GSDMD deficiency attenuated cartilage degeneration, synovitis, and subchondral bone sclerosis in the murine PTOA model, findings that provide a rationale for translational studies in OA using FDA-approved drugs that interfere with GSDMD pore-forming activity.

## Supplementary Information

**Additional file 1: Figure S1.** GSDMD deficiency did not reduce joint swelling and osteolysis induced by STIA. Six-week-old mice were injected intraperitoneally with PBS (*Gsdmd*^*+/+*^: 4 females and 3 males; *Gsdmd*^*-/-*^: 4 females and 2 males) or K/BxN mouse serum (*Gsdmd*^*+/+*^: 3 females and 2 males; *Gsdmd*^*-/-*^: 4 females and 3 males) on day 0 and 2. (**A** and **B**) Ankle thickness was measured daily for 12 days with a digital caliper. No differences in joint swelling were noted between male and female mice.

**Additional file 2: Figure S2.** GSDMD expression in articular cartilage of OA patients, cleavage and mediation of IL-1β and LDH release in murine macrophages. (**A**) qPCR analysis of GSDMD expression in articular cartilage of OA patients. Immunoblotting analysis of GSDMD cleavage (**B**) and IL-1β and LDH release in murine macrophages (**C**, **D**). *Gsdmd*^*+/+*^ and *Gsdmd*^*-/-*^ macrophages were primed with 100 ng/ml LPS or 30 ng/ml TNF-α for 3 h and treated with 15 μM nigericin for 1 hour. Whole-cell lysates were analyzed by immunoblot assay. IL-1β and LDH levels were measured in conditioned media. Data are representative of at least 3 independent experiments run in technical replicates and are mean ± SD. ***, *p* < 0.001; ****, *p* < 0.0001.

**Additional file 3: Figure S3.** Cartilage degeneration and subchondral bone sclerosis in MLI mice. Twelve-week-old *Gsdmd*^*+/+*^ and *Gsdmd*^*-/-*^ male mice were subjected to sham or MLI surgery. (**A**) OARSI scoring was performed to quantify the severity of OA. Two-way repeated measurement ANOVA analysis was used to determine the interaction effect*.* (**B**) Subchondral bone BV/TV was measured by μCT to quantify the extent of subchondral bone sclerosis. Two-way repeated measurement ANOVA analysis was used to determine the interaction effect*. N* = 7-8/group. Data are mean ± SD. **, *p* < 0.01; ***, *p* < 0.001.

**Additional file 4: Figure S4.** Effects of IL-1β on GSDMD expression in articular cartilage chondrocytes. Primary articular chondrocytes were treated with 1 ng/ml IL-1β for 24 hours. Whole-cell lysates were used for immunoblotting analyze GSDMD expression. β-actin was used as a loading control.

**Additional file 5.**

## Data Availability

All data generated or analyzed during this study are included in this published article [and its supplementary information files].

## Abbreviations

GSDMD: Gasdermin D
GSDME: Gasdermin E
IL-1: Interleukin-1
LDH: Lactate dehydrogenase
MLI: Meniscus ligamentous injury
OA: Osteoarthritis
PTOA: Post-traumatic osteoarthritis
RA: Rheumatoid arthritis
RANKL: Receptor activator of NF-κB ligand
STIA: Serum transfer-induced arthritis
