# Supplementary figures and images for: Gasdermin D deficiency attenuates arthritis induced by traumatic injury but not autoantibody-assembled immune complexes
Source: Arthritis Res Ther. 2021 Nov 16;23:286. doi: 10.1186/s13075-021-02668-8 (PMC8594229; doi:10.1186/s13075-021-02668-8)

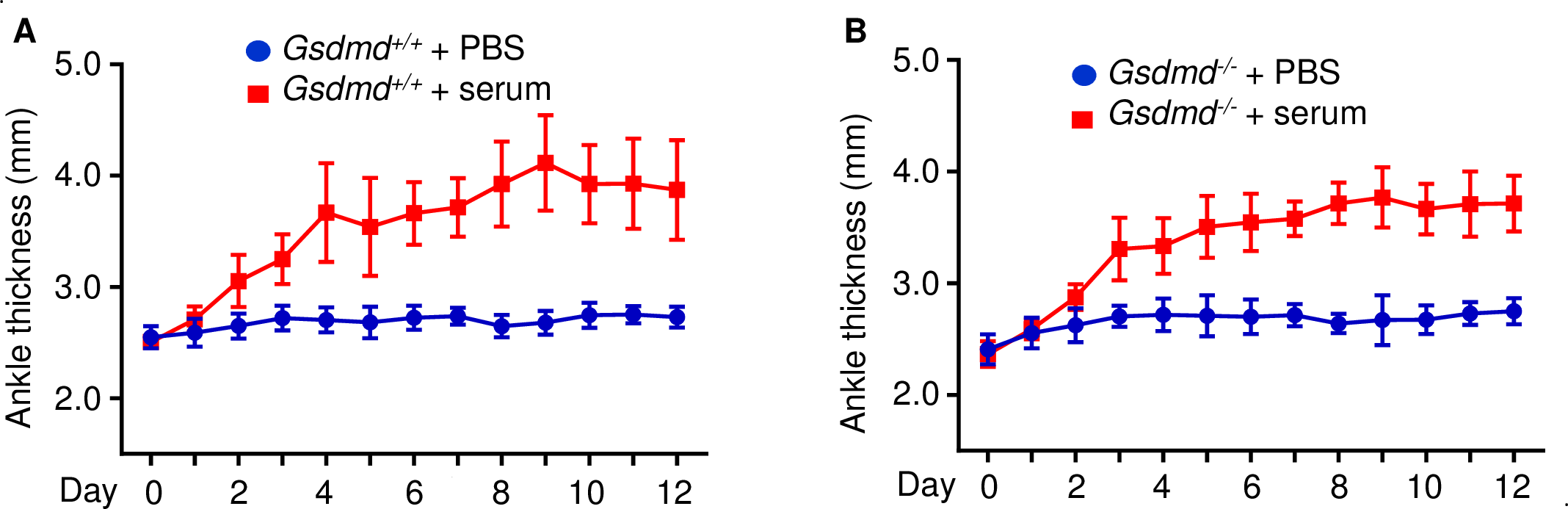

Supplement: Supplementary file 1 — Additional file 1: Figure S1. GSDMD deficiency did not reduce joint swelling and osteolysis induced by STIA. Six-week-old mice were injected intraperitoneally with PBS (Gsdmd+/+: 4 females and 3 males; Gsdmd-/-: 4 females and 2 males) or K/BxN mouse serum (Gsdmd+/+: 3 females and 2 males; Gsdmd-/-: 4 females and 3 males) on day 0 and 2. (A and B) Ankle thickness was measured daily for 12 days with a digital caliper. No differences in joint swelling were noted between male and female mice. [file 13075_2021_2668_MOESM1_ESM.tif]

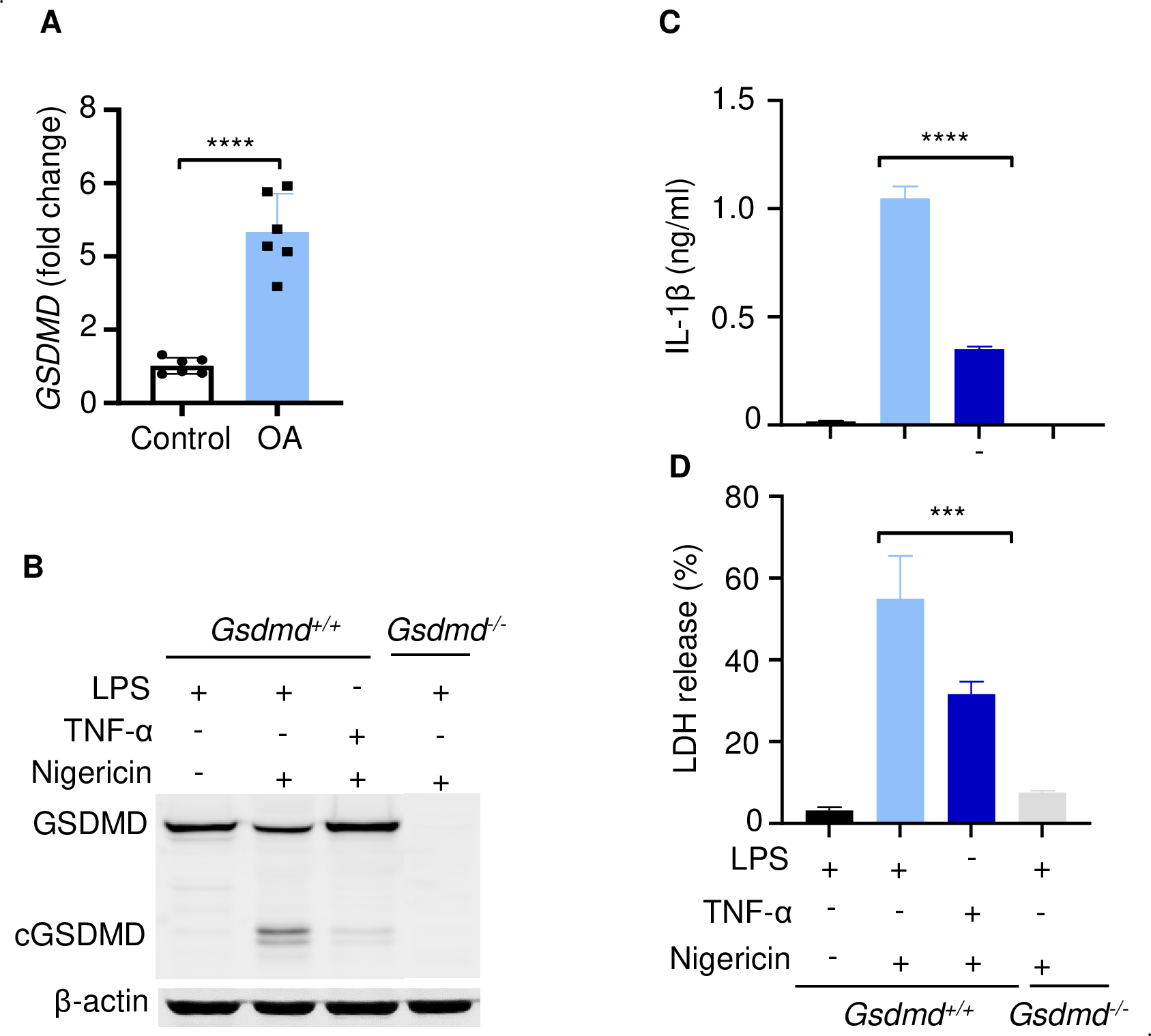

Supplement: Supplementary file 2 — Additional file 2: Figure S2. GSDMD expression in articular cartilage of OA patients, cleavage and mediation of IL-1β and LDH release in murine macrophages. (A) qPCR analysis of GSDMD expression in articular cartilage of OA patients. Immunoblotting analysis of GSDMD cleavage (B) and IL-1β and LDH release in murine macrophages (C, D). Gsdmd+/+ and Gsdmd-/- macrophages were primed with 100 ng/ml LPS or 30 ng/ml TNF-α for 3 h and treated with 15 μM nigericin for 1 hour. Whole-cell lysates were analyzed by immunoblot assay. IL-1β and LDH levels were measured in conditioned media. Data are representative of at least 3 independent experiments run in technical replicates and are mean ± SD. ***, p < 0.001; ****, p < 0.0001. [file 13075_2021_2668_MOESM2_ESM.tif]

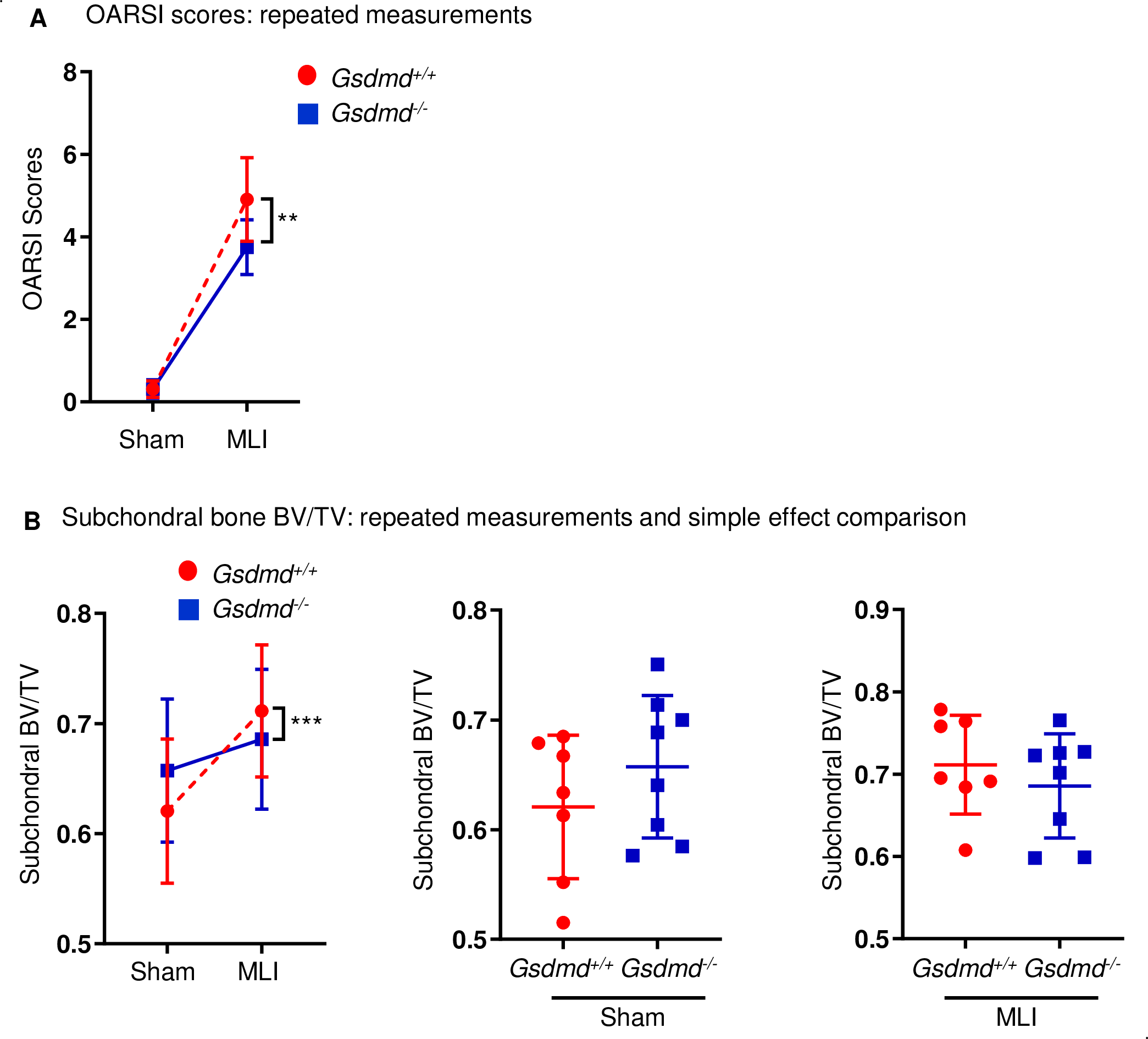

Supplement: Supplementary file 3 — Additional file 3: Figure S3. Cartilage degeneration and subchondral bone sclerosis in MLI mice. Twelve-week-old Gsdmd+/+ and Gsdmd-/- male mice were subjected to sham or MLI surgery. (A) OARSI scoring was performed to quantify the severity of OA. Two-way repeated measurement ANOVA analysis was used to determine the interaction effect. (B) Subchondral bone BV/TV was measured by μCT to quantify the extent of subchondral bone sclerosis. Two-way repeated measurement ANOVA analysis was used to determine the interaction effect. N = 7-8/group. Data are mean ± SD. **, p < 0.01; ***, p < 0.001. [file 13075_2021_2668_MOESM3_ESM.tif]

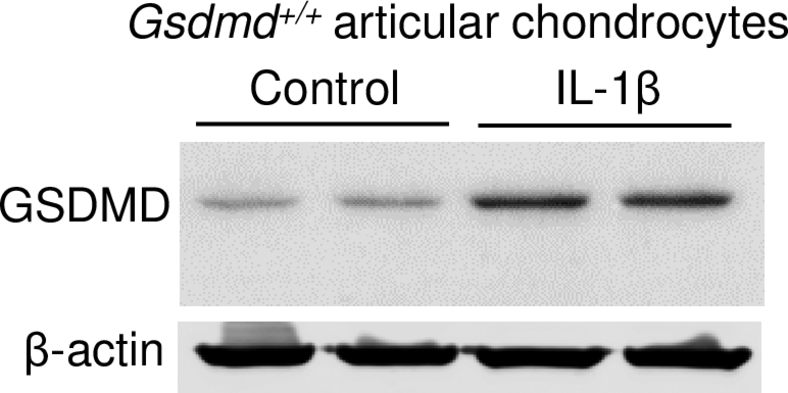

Supplement: Supplementary file 4 — Additional file 4: Figure S4. Effects of IL-1β on GSDMD expression in articular cartilage chondrocytes. Primary articular chondrocytes were treated with 1 ng/ml IL-1β for 24 hours. Whole-cell lysates were used for immunoblotting analyze GSDMD expression. β-actin was used as a loading control. [file 13075_2021_2668_MOESM4_ESM.tif]
