# Supplementary material for: Gasdermin D deficiency attenuates arthritis induced by traumatic injury but not autoantibody-assembled immune complexes
Source: Arthritis Res Ther. 2021 Nov 16;23:286. doi: 10.1186/s13075-021-02668-8 (PMC8594229; doi:10.1186/s13075-021-02668-8)
Supplement: Supplementary file 5 — Additional file 5. [file 13075_2021_2668_MOESM5_ESM.docx]

**Supplementary Table 1 (primers)**

| **Gene** | **Forward primer** | **Reverse primer** |
| --- | --- | --- |
| *Gsdmd (mouse)* | CCCGTTATTCATGTGTCAACCT | TGCCCTGAATGTTCCCATC |
| *GSDMD (human)* | GACCCTAACACCTGGCAGAC | CACCTCAGTCACCACGTACAC |
| *Gsdme (mouse)* | TGCAACTTCTAAGTCTGGTGACC | CTCCACAACCACTGGACTGAG |
| *IL1B (human)* | CTG AGC TCG CCA GTG AAA TG | TGT CCA TGG CCA CAA CAA CT |
| *IL18 (human)* | CAACAAACTATTTGTCGCAGGA | CAAAGTAATCTGATTCCAGGTTTTC |
